# Severe COVID-19 Pneumonia and Genetic Susceptibility: A Case Report and Literature Review

**DOI:** 10.7759/cureus.23636

**Published:** 2022-03-30

**Authors:** Badr A Alsayed, Rashid Mir

**Affiliations:** 1 Internal Medicine, University of Tabuk, Tabuk, SAU; 2 Molecular Oncology, Genetics and Translational Medicine, Faculty of Applied Medical Sciences, Tabuk, SAU

**Keywords:** covid-19, gene polymorphism, glutathione s-transferase, genetic susceptibility, sars-cov-2

## Abstract

Genetic susceptibility to severe acute respiratory syndrome coronavirus 2 (SARS-CoV-2) morbidity and mortality continues to evolve. This report presents a case of an apparently healthy male adult who developed severe coronavirus disease 2019 (COVID-19) and a study on relevant genetic mutations, namely, angiotensin-converting enzyme 2 (ACE2-rs4646994 I/D) gene, glutathione S-transferase (GST) M1 and T1 gene, and miR-423 rs6505162 C>A gene polymorphism. Results showed that the ACE-DD genotype of ACE2, (GSTM1+/+) (GSTT1−/−) genotype of GST gene, and CA genotype (heterozygosity) of miR-423 rs6505162 genes, which were found in the patient, could be independent risk factors of severe COVID-19, even without comorbidities.

## Introduction

Older patients and patients with comorbidities carry a higher risk for severe coronavirus disease 2019 (COVID-19) morbidity and mortality [1]. However, younger age groups, who generally have no chronic illnesses, may also suffer significantly from severe COVID-19 and its complications. COVID-19 outcomes vary between countries and different ethnicities; hence, researchers are encouraged to conduct genetic studies to explain this observation. Angiotensin-converting enzyme 2 (ACE2-rs4646994 I/D) gene, glutathione S-transferase (GST) M1 and T1 gene, and miR-423 rs6505162 C>A gene polymorphism may play a role in the host’s immune response to severe acute respiratory syndrome coronavirus 2 (SARS-CoV-2) [1-3]. Regarding disease management, portable chest x-ray (CXR) may be considered in the initial evaluation to minimize cross-infection risk and to avoid disrupting radiological services availability; however, it is an insufficient diagnostic tool for complicated COVID-19 cases [4]. The treatment dose of anticoagulation has been recommended for severe COVID-19, but it has risk factors that should be addressed. 

## Case presentation

**Figure 1:**
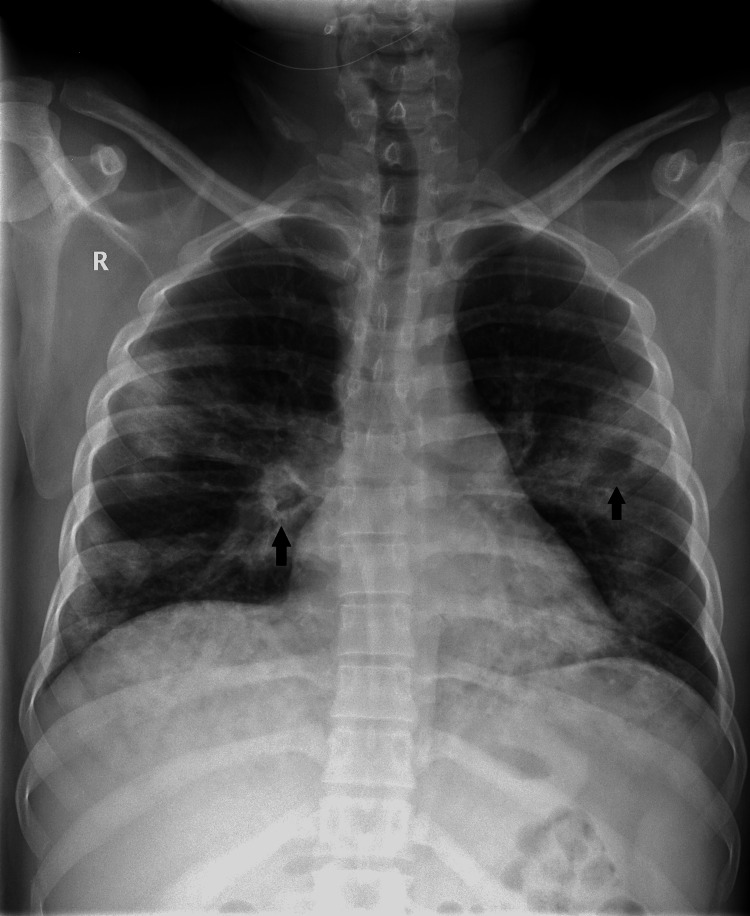
Chest x-ray shows bilateral parahilar and peripheral-based opacities, cavity-like radiolucent lesions (black arrows).

A 29-year-old male of Asian descent presented to our ER on April 22, 2021, complaining of fever, cough, and progressive dyspnea for the past five days. He worked as a blacksmith, did not smoke, and had no known chronic illnesses. Physical examination revealed a fair physique (BMI, 26 kg/m2), mild respiratory distress (22 breaths/minute), fever (38.5°C), and low normal oxygen saturation (O2Sat) (92% on ambient air); heart rate and blood pressure remained within the normal range (95 beats/minute, 101/56 mmHg). In chest examination, equal bilateral air entry with diffuse bronchial breathing and inspiratory crackles was found. Other systemic examinations were unremarkable. A reverse-transcriptase polymerase chain reaction (RT-PCR) assay from a nasopharyngeal swab detected SARS-CoV-2 RNA. Laboratory results were as follows: total white blood cell count, 6.5 × 10⁹/L; hemoglobin, 15.8 g/dL; platelet, from 154 × 10⁹/L to 103 × 10⁹/L; D-dimer, 1650 ng/mL, creatinine kinase (CK), 2420 IU/L; creatinine kinase-myocardial band (CK-MB), 43 IU/L; lactate dehydrogenase, 994 U/L; and total bilirubin, 107 μmol/L (mainly indirect). Arterial blood gas results were the following: pH, 7.347; partial pressure of arterial oxygen (PaO₂), 66 mm Hg; partial pressure of carbon dioxide, 47.5 mm Hg; and bicarbonate (HCO₃), 22 mEq/L. Additionally, electrocardiography detected sinus rhythm. However, CXR showed bilateral opacities involving more than one lobe in each lung, with suspicious shadows of cavitary lesions (Figure 1). 

Thus, he was found to have a severe bilateral COVID-19 pneumonia with impending cytokine storm. He then started taking an anticoagulant as a treatment dose (subcutaneous enoxaparin, 40 mg twice a day). Unfortunately, two days later, he became more dyspneic (31 breaths/minute), could not maintain O2Sat, and needed a non-rebreathing mask with 100% oxygen. He started manifesting significant hemoptysis; hence, anticoagulation de-escalated to a prophylactic dose. He was then shifted to the ICU. Computed tomography pulmonary angiogram (CTPA), which can rule out acute pulmonary embolism, could not be performed because of technical reasons and infection control protocols. A workup for cavitary lung lesions was conducted, and the results were as follows: sputum samples negative for acid-fast bacilli, no growth in sputum culture, normal renal functions, and unremarkable autoimmune panel examination. Over five days, no massive hemoptysis occurred. Although he lost approximately 1.5 g of hemoglobin, he did not require any blood transfusion. Eventually, hemoptysis stopped. In line with the Saudi Ministry of Health COVID-19 management protocol [5], the patient received antiviral therapy (favipiravir) as loading and maintenance doses in addition to systemic hydrocortisone and prophylactic dose anticoagulation.

**Figure 2:**
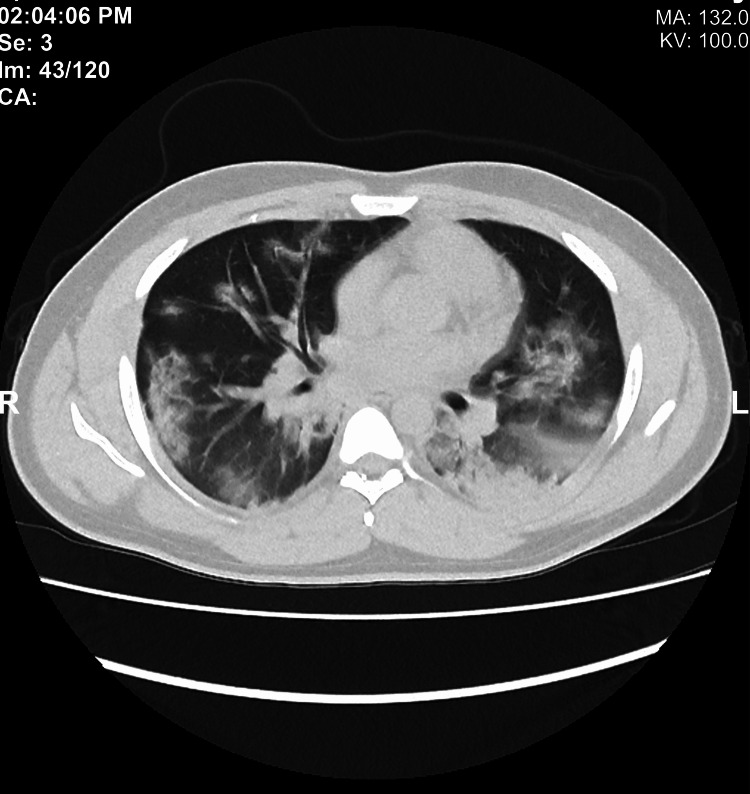
Chest HRCT shows bilateral peripheral ground-glass opacification and consolidation HRCT: high resolution computer tomography

Given the occurrence of hemoptysis after a therapeutic dose of anticoagulation followed by clinical stabilization with antiviral therapy, supportive measures, and prophylactic doses of anticoagulants, we deferred CTPA. High-resolution computed tomography (HRCT) of the chest showed a pattern of COVID-19 with overlapping shadows indicating CXR pseudo-cavitation findings (Figure 2). Three months after hospitalization, a follow-up CXR showed a complete resolution of COVD-19 consolidation.

Materials and methodology

Genomic DNA Extraction

A peripheral blood sample (2 ml) was collected in an ethylenediaminetetraacetic acid (EDTA) or Lavender top tube from the patient. The specimen was immediately stored at -30°C until processed. DNA isolation was done using DNeasy® Blood & Tissue Cat. No. 69504 (QIAGEN, Hilden, Germany) using manufacturer’s instructions. Genomic DNA was isolated from the patient as well as eight healthy controls. All samples were subjected to integrity, purity, and quality check by determining the optical density (OD) at 260 nm and 280 nm using NanoDrop™ One spectrophotometer (Thermo Fisher Scientific, Waltham, Massachusetts, USA). The OD determined was 1.90 indicating a good quality DNA. 

Determination of Insertion-Deletion of Angiotensin-Converting Enzyme 2 (ACE2) Gene

ACE2 insertion-deletion polymorphism (ACE2-rs4646994I/D) was determined by optimizing mutation-specific PCR (MS-PCR). The primers were designed using the Primer3 software as depicted in Table 1. Similar MS-PCR primers were used previously by Mir et al. [6]. Genomic DNA (50 ng) was amplified in a reaction volume of 25 μL, which contained 25 pmol of each of the primers given in Table 1 and 12 μL from Promega® PCR Master Mix (2x) (Cat. No. M7502-US) (Promega Corporation, Madison, Wisconsin, United States) and volume were adjusted to a final volume of 25 µL by adding nuclease-free double-distilled water (ddH2O).

**Table 1:** PCR primers of ACE2-rs4646994 I/D gene polymorphism PCR: polymerase chain reaction; ACE2: angiotensin-converting enzyme 2

Direction		Primer Sequence	PCR Product	Annealing Temperature
PCR primers of ACE2-rs4646994 I/D gene polymorphism				
ACE-F	5′-CTGGAGACCACTCCCATCCTTTCT-3′		490 bp (II)	58 °C
ACE-R	5′-GATGTGGCCATCACATTCGTCAGAT-3′.		190 bp (DD)	

Finally, we added 2 µL of the patient’s DNA. The thermocycling conditions used were 95°C for eight minutes followed by 45 cycles of 95°C for 35 seconds, annealing temperature at 58°C for 40 seconds, and extension at 72°C for 43 seconds followed by the final extension at 72°C for 10 minutes. The PCR products were electrophoresed on a 2% agarose gel stained with ethidium bromide to visualize three patterns: I/I (490 bp fragment), D/D (190 bp fragment), and I/D (both 490 and 190 bp fragments). 

Multiplex PCR GST M1 and T1 Genotyping

GST M1 and T1 genotyping were performed by multiplex PCR. The primers were also used previously (Table 2) [7]. Patient and control genomic DNA (50 ng) was amplified in a reaction volume of 25 μL, which was composed of 25 pmol of each of the primers depicted in Table 2. Six primers were used, 12 μL from Promega PCR Master Mix (2x) (Cat. No. M7502-US) was added, and the final volume was adjusted to 25 µL by adding nuclease-free ddH2O. Finally, we added 2 µL of the DNA sample of the patient. Multiplex PCR was performed to detect the GSTT1 and GSTM1 presence in the genomic DNA samples of patients and healthy controls. The cycling conditions comprised a hot start at 95°C for 10 minutes, followed by 35 amplification cycles at 95°C for 35 seconds, 59°C for 40 seconds, and 72°C for 58 seconds followed by one elongation step at 72°C for 10 minutes and storage at 4°C.

**Table 2:** Glutathione S-transferase (GST) M1 and T1 primers PCR: polymerase chain reaction

Gene	Primer sequence	Annealing temperature	PCR products
GSTM1	F-5′-GAACTCCCTGAAAA GCTAAAGC-3′	58˚C	215 bp
	R-5′-GTTGGGCTCAAATA TACGGTGG-3′		
GSTT1	F-5′-TTCCTTACTGGTCCT CACATCTC-3′		480 bp
	R-5′-TCACGGGATCATGGCC AGCA-3′		
CYP1A1	F-5′-GAACTGCCACTTCAGCTGTCT-3′		312 bp
	R-5′-CAGCTGCATTTGGAAGTGCTC-3′		

Gel Electrophoresis

The PCR products were separated by agarose electrophoresis through a 2% agarose and then visualized on the UView™ Mini Transilluminatoin (Bio-Rad Laboratories, Inc., Hercules, California, United States). The amplification of GSTM1 and GSTT1 primers yielded 215 bp and 480 bp PCR products, respectively, along with a 312 bp product for CYP1A1, which acted as an internal control for DNA integrity. The amplification of GSTT1/M1 genotypes is classified as either positive (when at least one GSTM1/T1 copy of the gene was present) or null. The homozygous and heterozygous characteristics for GSTT1 are represented as GSTT1 +/0 and GSTT1 +/+, respectively, and that for GSTM1 are represented as GSTM1 +/0 and GSTM1 +/+, respectively. 

miR-423 (rs6505162C>A) Genotyping

T-ARMS-PCR (amplification-refractory mutation system PCR) was utilized for the detection of miR-423 genotyping (rs6505162C>A). The primers were used as previously described and depicted in Table 3. Genomic DNA of the patient (50 ng) was amplified in a 24 μL ARMS PCR mixture containing 20 pmol of each of the mentioned primers and 12 μL from DreamTaq Green PCR Master Mix (2x) (Cat. No. K1081) (Thermo Fisher Scientific, Waltham, Massachusetts, United States). A final volume of 24ul was adjusted by adding ddH2O. Finally, 2 µL of the patient’s DNA was added. The PCR conditions were 95°C for eight minutes, followed by 38 cycles of 94°C, 60°C, and 72°C for 58, 25, and 45 seconds, respectively; and the final extension was performed at 72°C for five minutes. The gel was visualized on a gel documentation system, Gel Doc XR+ System (Bio-Rad Laboratories, Inc., Hercules, California, United States). The miR-423 PCR amplification products were separated by electrophoresis through a 2.5% agarose gel stained with ethidium bromide. The gel was visualized on the gel documentation system, Gel Doc XR+ System. Primers FI and RO amplified the C allele, which yielded a 160 bp band, whereas the same primers yielded a band of 228 bp from the T allele. Outer primers FO and RO yielded a 336 bp band, which acted as a control for DNA purity. 

**Table 3:** ARMS primers of miR-423 genotyping (rs6505162 C>A) ARMS: amplification-refractory mutation system

ARMS primers of miR-423 genotyping (rs6505162C>A)			
miR-423 FO:	5′-TTTTCCCGGATGGAAGCCCGAAGTTTGA-3′	336 bp	62°C
miR-423 RO:	5′-TTTTGCGGCAACGTATACCCCAATTTCC-3′		
miR-423 FI (T allele):	5′-TGAGGCCCCTCAGTCTTGCTTCCCAA-3′	228 bp	
miR-423 RI (C allele)	5′-CAAGCGGGGAGAAACTCAAGCGCGAGG-3′	160 bp	

Results

ACE2-rs4646994 I/D

**Figure 3:**
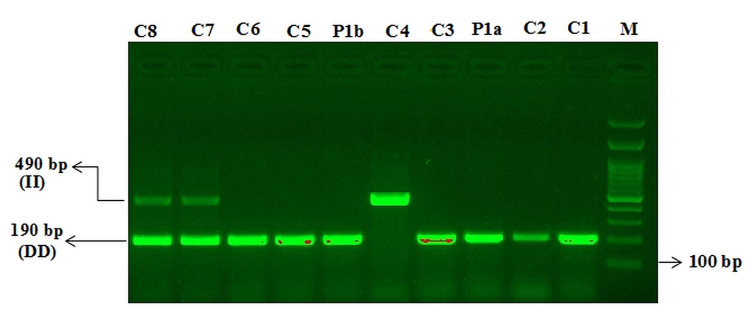
Mutation-specific PCR (MS-PCR) for the detection of insertion–deletion of ACE2-rs4646994 genotyping in controls and COVID-19 case report M: 100 bp DNA ladder DD genotype: C1, C2, P1a, C3, P1b, C5, C6 II genotype: C4 D/I genotype: C7, C8 COVID-19: coronavirus disease 2019; PCR: polymerase chain reaction; ACE2: angiotensin-converting enzyme 2

In the screening for ACE2-rs4646994 I/D gene polymorphism, DD genotype was found (Figure 3). 

GST M1 and T1 Genotyping

**Figure 4:**
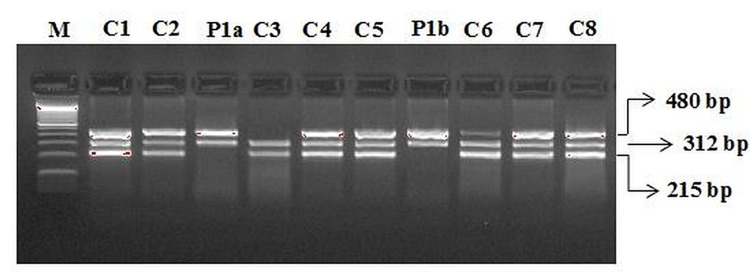
Multiplex PCR for the detection of glutathione S-transferase M1 and T1 genotyping controls and COVID-19 case report M: 100 bp DNA ladder GSTM1/T1: C1, C2, C4, C5, C6, C7, C8 GSTM1: C4 GSTT1: P1a, P1b COVID-19: coronavirus disease 2019; PCR: polymerase chain reaction

In the screening for GST M1 and T1 gene polymorphism, the GSTT1 genotype and a null genotype for GSTM1 were found (Figure 4).

miR-423 (rs6505162 C>A) Genotyping

**Figure 5:**
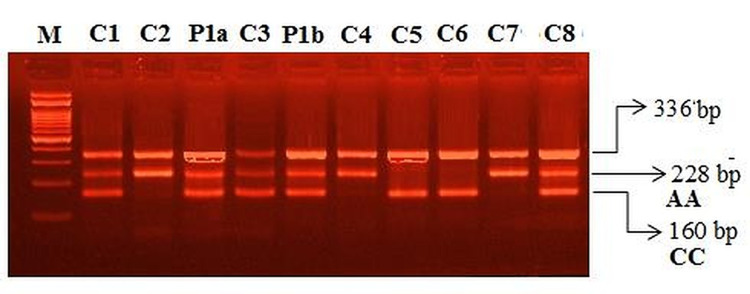
Amplification-refractory mutation system PCR (ARMS PCR) for the detection of miR-423 (rs6505162C>A) genotyping in controls and COVID-19 case report M: 100 bp DNA ladder MIR-423–homozygous (CC): C5, C6 MIR-423–homozygous (AA): C2, C4 & C7 MIR-423–heterozygous (C/A): C1, P1a, C3, P1b & C8

In the screening for miR-423 rs6505162 C>A gene polymorphism, CA genotype was found (Figure 5).

## Discussion

Severe COVID-19 in adults is characterized by dyspnea (≥30 cycles/minute), low blood oxygen saturation (≤93%), low ratio of partial pressure of oxygen (PaO2) to the fraction of inspired oxygen (FiO2) (<300), and/or high lung infiltrates (>50%) within 24-48 hours [8]. Similar to our case, severe illness usually begins one week after symptom onset, with dyspnea as the most common. Chronic obstructive lung disease, cardiovascular disease, and hypertension are among the most common predictive comorbidities for both severe disease and ICU admission, but our patient had none. However, being a male is one of the risk factors for severe COVID-19 [9]. Association between COVID-19 and pulmonary embolism creates a diagnostic and management challenge, especially in patients with known renal complications of COVID-19. Our patient had no hemoptysis at presentation in the ER, and his symptoms started after a therapeutic dose of anticoagulation. Therefore, a conservative approach was considered. However, hemoptysis accompanied with cavitary lung lesions shown in the baseline CXR suggests other differential diagnoses, mainly pulmonary tuberculosis, Streptococcus pneumoniae infection, Haemophilus influenzae infection, and Klebsiella pneumoniae infection [10]. HRCT confirmed the patient’s COVID-19 radiological pattern and ruled out cavitary lung lesions seen in the initial plain CXR. Bilateral peripheral ground-glass opacification with occasional consolidation is reportedly the marked CT feature of COVID-19 with no reported lung cavitation [11]. In the initial evaluation, portable CXR may be considered to minimize the cross-infection risk and to avoid disrupting radiological services availability [4]. In our case, depending solely on CXR was confusing and somewhat misleading. The CT patterns in COVID-19 are reportedly concordant with the PCR results; patients in whom CT shows greater parenchymal involvement are more likely to require ventilator support.

ACE2-rs4646994 I/D

The ACE1-DD genotype and D allele in the presence of diabetes and hypertension are significantly higher in patients with severe COVID-19 [6]. The ACE2-DD genotype is linked with increased COVID-19 mortality; hence, genotyping for ACE1 I/D polymorphism could be used to assess risk and predict severity to achieve better COVID-19 prognosis and management [1]. Furthermore, the ACE2 polymorphism is associated with the varying degrees of disease severity and clinical outcomes of COVID-19, with the absence of ACE-DD genotype conferring protection against severe lung injury. In the United States, African Americans with a high frequency of the D allele have higher mortality rates [12].

Role of GSTM1 and GSTT1 genes

Several studies reported that deletions in the GSTT1 (GSTT1 −/−) and GSTM1 (GSTM1 −/−), also called null genotypes, are linked with the loss of enzyme activity of the GST gene, increasing the risk for oxidative stress associated with multifactorial diseases including cardiovascular and respiratory diseases [13].

In addition, males with GSTT1−/− alone or in combination with GSTM1−/− genotype had an excessive decrease in forced expiratory volume in the first and second forced expiratory volume (FEV1) regardless of the smoking status. Abbas et al. reported that the frequency of GSTM1−/−, GSTT1−/−, and GSTM1−/−/GSTT1−/− was higher in severe COVID-19 than in mild COVID-19, and patients with both GSTM1+/+ and GSTT1−/− genotypes showed a poor survival rate (p = 0.02); therefore, patients with COVID-19 having the GSTT1−/− genotype had a high mortality rate [14]. Ding et al. showed that the presence of GSTT1−/− and/or GSTM1−/− showed a higher risk of developing pulmonary fibrosis in patients with chronic obstructive pulmonary disease, which is also one of the most important complications of COVID-19 and characterized by long-term respiratory complications [15]. In previous results, patients with COVID-19 having the GSTT1−/− genotype showed higher mortality. The main observation of our genetic study is that the patient was carrying a null genotype for GSTT1 (GSTT1−/−) but was carrying a GSTM1 genotype (GSTM1+/+). Therefore, our patient was classified as having “(GSTM1+/+) (GSTT1−/−).” Saadat reported that (GSTM1+/+) (GSTT1−/−) was positively associated with COVID-19 mortality but had no correlation with COVID-19 prevalence; individuals with a lower frequency of the GSTT1-null genotype exhibit higher COVID-19 mortality [2]. We should also consider that GST genotype distribution can be ethnicity-dependent. Recently, it has been reported that the GSTP1-Ile105Val gene polymorphism is associated with increased morbidity and mortality in patients with COVID-19 and a higher incidence of GSTP1-Ile105Val gene polymorphism is directly proportional to mortality in different countries [2].

Role of microRNAs (miRNAs)

Mature miRNAs are a class of naturally occurring, small, non-coding RNA molecules, about 21-25 nucleotides in length. MiRNAs are partially complementary to one or more messenger RNA (mRNA) molecules, and their main function is downregulating gene expression in a variety of manners, including translational repression, mRNA cleavage, and deadenylation. Mature microRNAs play an essential role in controlling the immune system function and are associated with the pathogenesis and etiology of many types of disorders [3,16]. Mature miRNAs are involved in the immunological responses to microbial and viral respiratory infections, including respiratory syncytial virus and influenza virus, and there is a significant correlation with the ectopic expression of mature miRNAs. It has been reported that any deregulation of the mature miRNA's expression in the epithelial cell can lead to the pathogenesis of chronic and acute respiratory infections. Thus, elucidating the mechanisms of the mature miRNAs in the immunological responses and characterizing the mature miRNA's target genes could help understand the molecular mechanisms between viruses and hosts. In turn, this could help to understand therapeutic strategies for preventing and treating acute COVID-19 infections [17-19].

Distinct miRNA profiles were also observed in patients with COVID-19 requiring oxygenation. SARS-CoV-2 infection induces a robust host miRNA response that could improve COVID-19 detection and patient management [16]. Numerous disorders exhibit the abnormal expression of both mature forms of miRNA-423-5p and miR-423-3p [19,20]. Recently, diabetes, obesity, and hypertension had shown downregulation of the circulating miRNA-146a, which may be due to increased inflammation, fibrosis, and the systemic effects accompanying severe COVID-19 [20].

## Conclusions

Our patient, who was young and previously healthy, developed severe bilateral COVID-19 pneumonia and carried the ACE DD genotype of ACE2, (GSTM1+/+) (GSTT1−/−) genotype of GST gene, and CA genotype (heterozygosity) of MIR423 rs6505162 gene. ACE DD genotype is strongly associated with increased disease severity of COVID-19 and is also linked with high COVID-19 mortality. GSTM1 (GSTM1+/+) (GSTT1−/−) null genotype is significantly associated with a high risk for disease severity and poor survival rate. Therefore, our case report with genetic studies might encourage researchers to conduct further studies to identify populations susceptible to severe COVID-19 for targeted interventions, even in seemingly healthy individuals.

## References

[1] Verma S, Abbas M, Verma S (2021). Impact of I/D polymorphism of angiotensin-converting enzyme 1 (ACE1) gene on the severity of COVID-19 patients. Infect Genet Evol.

[2] Saadat M (2020). An evidence for correlation between the glutathione S-transferase T1 (GSTT1) polymorphism and outcome of COVID-19. Clin Chim Acta.

[3] Li C, Hu X, Li L, Li JH (2020). Differential microRNA expression in the peripheral blood from human patients with COVID-19. J Clin Lab Anal.

[4] Jacobi A, Chung M, Bernheim A, Eber C (2020). Portable chest X-ray in coronavirus disease-19 (COVID-19): A pictorial review. Clin Imaging.

[5] (2021). Saudi MoH Protocol for Patients Suspected of/Confirmed with COVID-19 ... or confirmed COVID-19 infection. (2021). Accessed: 22/1/2022. Saudi MoH Protocol for Patients Suspected of/Confirmed with COVID-19: Supportive Care And Antiviral Treatment Of Suspected Or Confirmed Covid-19 Infection.

[6] Mir MM, Mir R, Alghamdi MA, Alsayed BA, Wani JI, Alharthi MH, Al-Shahrani AM (2021). Strong association of angiotensin converting enzyme-2 gene insertion/deletion polymorphism with susceptibility to SARS-CoV-2, hypertension, coronary artery disease and COVID-19 disease mortality. J Pers Med.

[7] Abu-Duhier F, Mir R (2017). GSTT1 (rs4025935) null genotype is associated with increased risk of sickle cell disease in the populations of Tabuk-Northwestern region of Saudi Arabia. Hematology.

[8] Wu Z, McGoogan JM (2020). Characteristics of and important lessons from the coronavirus disease 2019 (COVID-19) outbreak in China: summary of a report of 72 314 cases from the Chinese Center for Disease Control and Prevention. JAMA.

[9] Jain V, Yuan JM (2020). Predictive symptoms and comorbidities for severe COVID-19 and intensive care unit admission: a systematic review and meta-analysis. Int J Public Health.

[10] Ammar A, Drapé JL, Revel MP (2021). Lung cavitation in COVID-19 pneumonia. Diagn Interv Imaging.

[11] Xu Z, Pan A, Zhou H (2020). Rare CT feature in a COVID-19 patient: cavitation. Diagn Interv Radiol.

[12] Dyer O (2020). Covid-19: black people and other minorities are hardest hit in US. BMJ.

[13] Allocati N, Masulli M, Di Ilio C, Federici L (2018). Glutathione transferases: substrates, inihibitors and pro-drugs in cancer and neurodegenerative diseases. Oncogenesis.

[14] Abbas M, Verma S, Verma S (2021). Association of GSTM1 and GSTT1 gene polymorphisms with COVID-19 susceptibility and its outcome. J Med Virol.

[15] Ding Z, Wang K, Li J, Tan Q, Tan W, Guo G (2019). Association between glutathione S-transferase gene M1 and T1 polymorphisms and chronic obstructive pulmonary disease risk: A meta-analysis. Clin Genet.

[16] Farr RJ, Rootes CL, Rowntree LC (2021). Altered microRNA expression in COVID-19 patients enables identification of SARS-CoV-2 infection. PLoS Pathog.

[17] Pinilla L, Benitez ID, González J, Torres G, Barbé F, de Gonzalo-Calvo D (2021). Peripheral blood microRNAs and the COVID-19 patient: methodological considerations, technical challenges and practice points. RNA Biol.

[18] Wang Y, Zhu X, Jiang XM (2021). Decreased inhibition of exosomal miRNAs on SARS-CoV-2 replication underlies poor outcomes in elderly people and diabetic patients. Signal Transduct Target Ther.

[19] Jha CK, Mir R, Elfaki I (2019). Potential impact of microRNA-423 gene variability in coronary artery disease. Endocr Metab Immune Disord Drug Targets.

[20] Mu X, Wang H, Li H (2021). Silencing of long noncoding RNA H19 alleviates pulmonary injury, inflammation, and fibrosis of acute respiratory distress syndrome through regulating the microRNA-423-5p/FOXA1 axis. Exp Lung Res.

